# Sars-Cov2 world pandemic recurrent waves controlled by variants evolution and vaccination campaign

**DOI:** 10.1038/s41598-022-22816-7

**Published:** 2022-10-27

**Authors:** Gaetano Campi, Andrea Perali, Augusto Marcelli, Antonio Bianconi

**Affiliations:** 1 Institute of Crystallography, Consiglio Nazionale delle Ricerche CNR, Via Salaria Km 29.300, Monterotondo Roma, 00015 Rome, Italy; 2 Rome International Centre Materials Science, Superstripes RICMASS, Via dei Sabelli 119A, 00185 Rome, Italy; 3 Physics Unit, School of Pharmacy, University of Camerino, 62032 Camerino, MC Italy; 4 INFN-Laboratori Nazionali di Frascati, Via E. Fermi 54, 00044 Frascati, RM Italy; 5 National Research Nuclear University MEPhI (Moscow Engineering Physics Institute), Moscow, Russian Federation 115409

**Keywords:** Biophysics, Physics

## Abstract

While understanding the time evolution of Covid-19 pandemic is needed to plan economics and tune sanitary policies, a quantitative information of the recurrent epidemic waves is elusive. This work describes a statistical physics study of the subsequent waves in the epidemic spreading of Covid-19 and disclose the frequency components of the epidemic waves pattern over two years in United States, United Kingdom and Japan. These countries have been taken as representative cases of different containment policies such as "Mitigation" (USA and UK) and "Zero Covid" (Japan) policies. The supercritical phases in spreading have been identified by intervals with RIC-index > 0. We have used the wavelet transform of infection and fatality waves to get the spectral analysis showing a dominant component around 130 days. Data of the world dynamic clearly indicates also the crossover to a different phase due to the enforcement of vaccination campaign. In Japan and United Kingdom, we observed the emergence in the infection waves of a long period component (~ 170 days) during vaccination campaign. These results indicate slowing down of the epidemic spreading dynamics due to the vaccination campaign. Finally, we find an intrinsic difference between infection and fatality waves pointing to a non-trivial variation of the lethality due to different gene variants.

## Introduction

The ongoing COVID-19 pandemic infection had its epicenter in Wuhan at the end of 2019; it later spread rapidly to China and other countries, with cases in Europe initially limited to small clusters in Germany, France and the UK. On February 20, 2020, the first case of locally acquired SARS-CoV-2 infection in Northern Italy was diagnosed. In the two subsequent years, the pandemic has spread, giving rise to several variants and mutations that have caused millions of cases with the most disparate symptoms, from severe pneumonia that has led to hundreds of thousands of deaths worldwide, to complete asymptomaticity. Nowadays, understanding the time evolution of the new viral quasispecies of SARS-CoV-2 appearing during Covid-19 pandemic spreading^1–7^ is an important challenge for the physics of life. Historically, pandemics diffusion is characterized by periodic traveling waves described by reaction diffusion equations^8–10^, by cyclic predator–prey models^11–13^ and diffusive periodic SIR epidemic models^14^.

In this work we provide the statistical physics analysis of recurrent waves in the complex world progression of Covid-19 pandemic over two years, related with the rate of gene variants of SARS-CoV-2^1–3^ competing with the natural immunity response and the vaccination campaign^15^. Mathematical models for the infection progression and the reaction–diffusion model of the viral evolution as determined by the competition of virus strains have been recently proposed^8,9,16^ and statistical physics theory has been used to understand the space and time spreading epidemics^17–20^. Actually, the pandemic Covid-19 progression after two years from the outbreak has been characterized mainly by prompt enforcement of containment mitigation measures^21–30^ in the first year and by both the appearance of new gene variants and the massive vaccination campaign in the second year. Indeed, the virus evolves in competition with the human immune response, which is modified by the vaccination campaign^3^, developing from early variants that are replaced time by time by emerging new variants^1,2^.

Data on a sizeable minimum time lapse evolution are now available after two years from the Covid-19 outbreak, which allows us in the first part of this work to study the recurrent waves in the Clovid-19 world pandemic and to compare it with previous world epidemics like Spanish flu and measles, which have shown periodic traveling waves^31–33^. In the second part we describe the spatial heterogeneity of the pandemic in different countries. In fact, the recurrent Covid-19 waves are different because of a) different political containment measures enforced in each country and b) time for the appearance of a new SARS-CoV-2 virus gene variant. As an example, in the beginning of 2021 the pandemic evolution has seen the emergence of the new virus gene variant B.1.1.7 called *alpha* (UK), with numerous mutations in its spike protein sequence. Its spreading rate was at least 50% faster than earlier circulating lineages. This variant was followed by the *beta* variant from South Africa, by the *gamma* variant from Brazil, by the *delta* variant from India^1,2^ up to the appearing at the end of 2021 of the *Omicron* variant, rapidly spreading all over the world. In order to investigate the spatial heterogeneity on different populations we have selected three countries from different continents: United Kingdom for Atlantic Europe, Japan for Pacific Asia and USA for America.

After the native species of Wuhan, a European variant named *20E* appeared in the UK around 08/10/2020. This variant did not spread to the USA and Japan, and gave way to the *alpha* variant around 25/12/2020. Subsequently, this *alpha* variant reached the USA and Japan around 25/02/2021 and 23/03/2021, respectively. The next major variant, *delta*, from India first reached the UK around 24/05/2021 and then the USA around 13/06/2021 and Japan around 21/07/2021.

The selected countries represent different containment policies enforced by different health policies. Japan has been selected as a case belonging to the group of countries as South Korea, China and Australia where the policy of "*Covid zero*"^21,22,30^ has been enforced. These countries have chosen to stop the number of new infections by strict lockdowns, borders control, case finding and mobile contact tracing. The case of USA and United Kingdom have been selected being representative of the "*Mitigation policy*" where lockdowns with loose mitigation rules have been enforced^7,21–30,34^. In addition to these non-pharmaceutical interventions, the spread of COVID-19 was thwarted by the distribution of COVID-19 vaccines, which began between the end of 2020 and the beginning of 2021. In particular, the vaccination campaign began around 13/12/2020 in the USA, around 10/01/2021 in the United Kingdom and subsequently, on 24/02/2021 in Japan. To date, as of 01/28/2022, the USA, UK and Japan have reached 75.5, 77.7 and 81.4% of the population, of fully vaccinated people (with 2 doses).

In this work we describe the evolution of Sars-Cov2 worldwide and in the three selected countries using the following time dependent parameters: (i) the *RIC* index, and the (ii) the *fatality rate*. The *RIC* index measures the recurrent waves of the infections by combining the evolution of two different key physical variables: the effective reproductive rate, R_t_, and the doubling time, T_d_^21^. *T*he *fatality rate (Df)* given by the daily deaths per million population, measures the severity of the disease. The spreading of the virus in the (*RIC*, *Df*, time) phase space draws a complex helicoid orbit made of periodic traveling waves. The orbit has been studied by Wavelet analysis to visualize the periodicity of spreading in the frequency domains and by the time evolution of Largest Lyapunov Exponents using a method suitable to deal with transient, non-deterministic and non-stationary short time series.

## Results

### Spreading of Sars-Cov2 worldwide

The pandemic evolution in epidemiology is typically monitored by the effective reproductive number, R_t_, which measures the potential transmissibility of the disease and therefore its contagiousness rate. In any case, R_t_ does not measure the rate of the recurrent waves of the pandemic agent and therefore it cannot provide a full description of the pandemic spreading vs. time. In fact, it may happen that a high R_t_ value, which generally describes a high transmissibility pathogen, can coexist with a low transmission speed that allows the epidemic spread to be kept under control. Thus we need to consider the speed of waves measured by the doubling time, T_d_, that is the time in which the number of infected individuals doubles, as extracted from the cumulative curves of the total cases.

**Figure 1:**
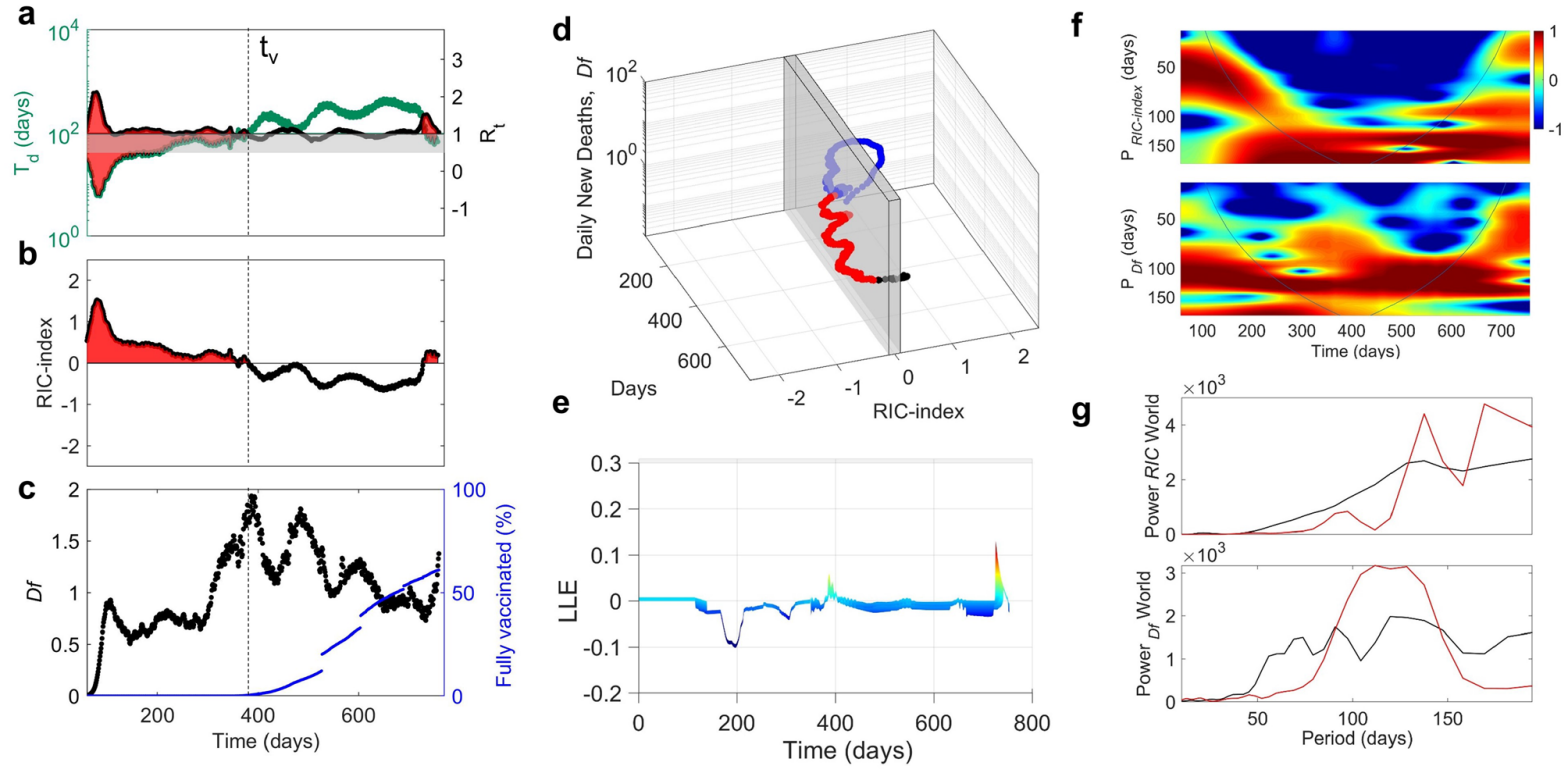
T_d_, R_t_, RIC-index and daily fatalities per million populations (*Df*) of the epidemic spreading in all worldwide from 15/03/2020 to 28/01/2022. (**a**) Plots of the time dependent doubling time, T_d_, (green dots) and reproductive rate R_t_ (black dots) in the world from 15/03/2020 to 28/01/2022. The gray region below R_t_ = 1 represents the *critical* region separating the *supercritical* (T_d_ < 50, R_t_ > 1) from the *subcritical* (T_d_ > 100, R_t_ < 1). The *supercritical* phases correspond to the red areas. The vaccination campaign starts at t = t_v_ = 350 day (dotted vertical line) corresponding to 15/12/2020. (**b**) The *RIC* index (black dots) vs. time. The *supercritical* phases occur for positive values of the *RIC* index, indicated by the red areas. (**c**) Daily new deaths per million of population (full circles) and percentage of fully vaccinated peoples (blue line) vs. time. (**d**) 3D orbit of the Covid-19 vortex over two years where the daily new deaths per million population and the *RIC-index* (days) are plotted as a function of time measured in days starting from the 75 day of the year 2020. The gray shaded plane represents the critical crossover at the zero *RIC* index, which separates the *supercritical RIC*-index > 0 and the *subcritical* regime *RIC* index < 0. (**e**) Lyapunov map showing the transition from convergent to divergent trajectories, i.e., the instability zones where the Larger Lyapunov Exponent (LLE) becomes positive. **(f)** Local wavelet power spectrum, obtained from the Wavelet transform of the *RIC* index and daily deaths per million population *(Df)* time series. We can observe the evolution of the main periods. We note a shift towards longer periods with the activation of the vaccination campaign in both *RIC* index and *Df* time series. We can also visualize short lived transient events during the pandemic spreading, corresponding to lower intensity spots at small periods. Power is color coded from − 1 (blue) to 1 (red). (**g**) The Global Wavelet Power Spectrum (GWPS) of the period of epidemic periodic traveling waves for infections measured by the *RIC* index and *Df* (solid black line), before the vaccination campaign for *t* < *t*_*v*_ (blue line) and after the onset of the vaccination campaign for *t* > *t*_*v*_ (red line).

In Fig. 1a we show the time dependent doubling time, T_d_, and the reproductive, R_t_, worldwide from 15/03/2020 to 28/01/2022. The expanded parametric space (T_d_, R_t_, t) allows to recognize and identify three phases: *supercritical*, *critical* and *subcritical*^21–24^. The *supercritical* phase, outline by red areas, is characterized by an exponential growth of infections with R_t_ > 1 and T_d_ < 50 days. In the *subcritical* phase, R_t_ is < 1, T_d_ becomes greater than 100 days and the pandemic is arrested. Between the *subcritical* and *supercritical* phases, we can define a critical regime highlighted by the gray stripe where 1 < R_t_ < 1.1 and 50 < T_d_ < 100 days, i.e., regions where metastable phases can occur^24^. In order to take into account both the reproductive rate and the rate of spread to describe the evolution of pandemics, we introduced a new parameter called the *RIC* index:

1 $$RIC = {\text{ log}}\left( {{\text{R}}_{{\text{t}}} /{\text{T}}_{{\text{d}}} } \right) - {2}$$

The R_t_ parameter gives transmissibility of the virus, while the doubling time, T_d_, takes into account the speed with which it transmits. This parameter is able to monitor the infections spreading, that is the contagiousness of the pandemic state by identifying: *i*) metastable phases precursors of subsequent pandemic waves when it approaches the zero value (*RIC* = 0), *ii*) the *supercritical* (*RIC* > 0) and *subcritical* (*RIC* < 0) phases for positive and negative values, respectively^21,23,24^. Figure 1b shows the *RIC* index from 15/03/2020 to 28/01/2022 worldwide. We can identify both *supercritical* phases (red areas with *RIC* > 0) and the subcritical phases (*RIC* < 0). We can observe how the *RIC* index falls below the critical value of zero after the beginning of the vaccination campaign for t > t_v_ = 350 day, although during the vaccination campaign there were further waves contained in the subcritical phase with different duration and intensity. A return in the supercritical zone occurred around the day 700, with the emergence of the last Covid mutation, the *Omicron*. Alongside the *RIC* index, which gives us a measure of the infections spreading, another important factor to consider in a pandemic is the severity of the disease. For this purpose, we used the fatality rate, that is the number of daily deaths per million population *(Df)*.

In Fig. 1c we report *Df* from 15/03/2020 to 28/01/2022 worldwide. Also here we observe the decreasing trend of the fatalities during the vaccination campaign for t > t_v_ and the increase of *Df* with the spreading of the *Omicron* variant. The blue line in Fig. 1c represents the percentage of fully vaccinated people.

### Worldwide evolution of severity versus contagiousness of the disease: helicoid vortex in (RIC, Df, t) space

The recurring waves in the world pandemic evolution can be visualized by plotting the daily number of deaths *Df* as a function of the *RIC* index and time, thus taking into account both the fatalities and the infections. As a result we obtain an orbit similar to a helicoid vortex, shown in Fig. 1d, typical of non-linear and chaotic phenomena. In non-linear systems, the chaotic character of a time series is generally determined by calculating the Lyapunov exponent on the reconstructed phase space, which tells us if the trajectory becomes unstable and unpredictable. If two initial states are separated by a distance *s*_0_, for a short time, the separation will evolve according to the equation s(*t*) ≈ *s*_0_*e*^λ*t*^. The value λ is defined as the Larger Lyapunov Exponent (LLE) of the system. Chaotic systems show positive Lyapunov exponents and their sensitivity to initial conditions increases exponentially with λ. Conversely, a negative Lyapunov exponent indicates that the orbit converges towards a stable fixed point, as generally occurs in a dissipative system with asymptotic stability. In stationary systems the phase space reconstruction usually requires long time series, however the Covid 19 epidemic is a transient phenomenon with short time series due to the emergence of unpredictable point mutations in the gene and to the competition with the immune system, which is itself time dependent. In order to analyze this non-deterministic evolution, we have used the Rosenstein^35^ method capable of estimating the LLE for transient and short time series. This method has been used and tested in a wide variety of non-linear phenomena such as biological time series characterized by transient states that diverge from classical chaotic systems^36–38^. In our case, the chaotic character in the pandemic evolution is described through Lyapunov maps obtained by calculating the variation of the LLE on 3D orbits representing the variation of *Df* vs. the *RIC* index. The detection of the crossover from negative to positive LLE allows to identify areas of instability with a chaotic and non-predictive character, which identifies the onset of new pandemic waves. In this phenomenon shown in Fig. 1e we observe three main time domains defined by the Lyapunov map for the world pandemic and three crossover points to be associated to the blue, red and black regions in the helicoid vortex of Fig. 1d. The blue and red trajectories correspond to the pandemic spreading before and after the start of the vaccination campaign. The last crossover on the map indicates the onset of the *supercritical* phase due to the *Omicron* variant.

### Worldwide recurrent waves of Covid-19: Wavelet Analysis

Finally, one of the most interesting results obtained in this work concerns the effects of the vaccination campaign on the pandemic waves in the space of temporal frequencies (or periods). This was obtained by applying the Wavelet Transform (WT) on the time series of both *RIC* index and daily deaths *Df*. In this way we have visualized the pandemic evolution by means of the components in Fourier space, as a function of time. The Local Wavelet Power Spectrum (LWPS) of the *RIC* index in Fig. 1f clearly shows a softening after t_v,_ i.e., the beginning of the vaccination campaign. Peaks in the LWPS identify the most prominent epidemic periods in the time series. Relevant unexpected differences among periods in the infection waves measured by the *RIC* index and the fatality rate periods measured by *Df* can be identified. The common feature in all spectra is a wide characteristic peak centered at 130 ± 20 days. Moreover, an increasing probability for a short (80 ± 20 days) and a long (170 ± 20 days) period in the infection waves after the vaccination can be observed. On the other hand, we observe a decreasing probability for the short (80 ± 20 days) and long (170 ± 20 days) period in the fatality waves after the vaccination, characterized instead by a broadening of the dominant period at 130 days. This key result of the analysis demonstrates that the fatality and infection waves evolve differently worldwide, a signature of the variation of the lethality of different variants. Therefore, fatality and infection waves are not related simply by a temporal shift, but exhibit a qualitative different spectrum of the periods. This is confirmed by the Global Wavelet Power Spectrum (GWPS) showed in Fig. 1g and obtained by the time averaging of the LWPS before and after t_v_. It provides information similar to the traditional Fourier analysis, but in different time windows.

### Severity of Sars-Cov2 spreading in United States, United Kingdom and Japan

The traveling pandemic waves in different countries can be controlled by different factors. Although the uncontrolled growth of the pandemic spread is known to follow the law of exponential growth, the epidemic spread of Covid-19 turned out to be different as it was modified by containment policies, tuned in time in order to avoid the saturation of health facilities and minimize the economic losses. The various containment measures changed the expected exponential growth in a type of frustrated growth as observed in various complex systems and materials and typical, p. ex., of controlled fission processes in nuclear reactors. Alongside the containment policies, the pandemic waves can be also perturbed by the emergence of new variants with a complex interplay of different factors. The phenomenon can be treated by applying the methodology shown above for the world case to three reference countries that adopted different containment policy measures: United States, United Kingdom and Japan, belonging to different geographical areas.

**Figure 2:**
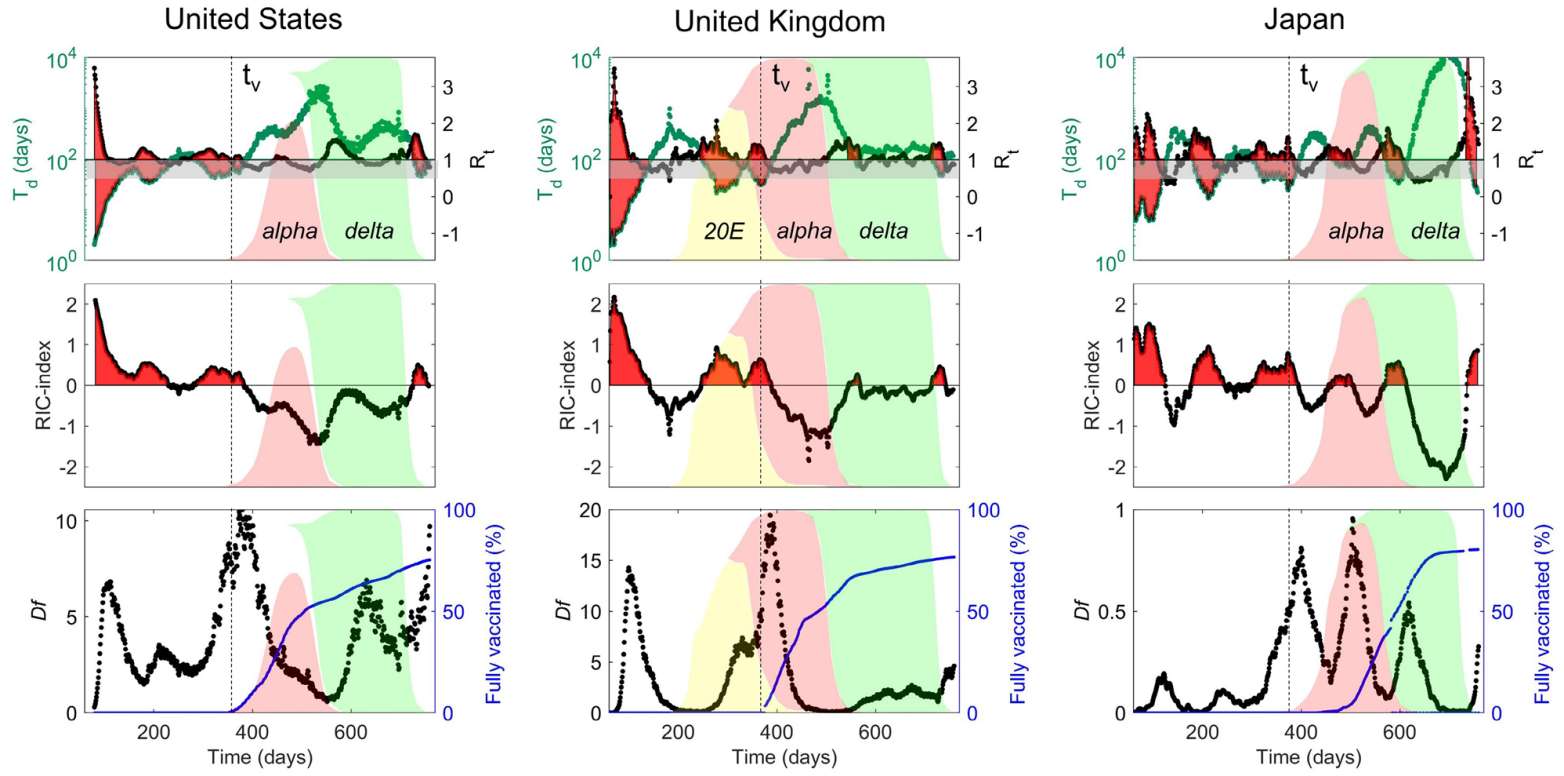
Epidemic spreading from 15/03/2020 to 28/01/2022 in United States, United Kingdom and Japan as described by T_d_, R_t_, RIC-index and daily fatalities per million populations (*Df*). The upper panels show plots of the time dependent doubling time^21^, T_d_, (green dots) and of the reproductive rate R_t_ (black dots). The red areas are the supercritical phases that correspond to T_d_ < 50 and R_t_ > 1. The gray shaded stripe represents the critical crossover from the supercritical to the subcritical phase with T_d_ > 100 and R_t_ < 1. The three middle panels report the evolution of the *RIC* index where the horizontal black line separates the *supercritical RIC* index > 0 and the *subcritical* regime *RIC* index < 0. In the three lower panels we show the number of daily new deaths *(Df)* and the percentage of fully vaccinated people. In all panels the vertical dashed black line at t = t_v_ = 350 (15/12/2020) indicates the beginning of the vaccination campaign. We show also the intervals associated with the recurrent waves of the different coronavirus variants (colored areas) indicating the proportion of total number of sequences, over time, that fall into defined variant groups. The top of the colored areas on the Y-axis correspond to '1'. Sequence counts are binned into 2-week intervals. In the white area before the vaccination campaign the prevalent variant is the Wuhan virus strain. After t_v_ the European 20E, *Alpha*, and *Delta* variants are reported. Finally, the last *Omicron* spreads in the white regions after the *Delta* light green areas.

In Fig. 2 we show the pandemic evolution vs. time of T_d_, R_t_, *RIC* index and *Df*. As in Fig. 1, we highlighted *supercritical* and *critical* phases with red zones and gray stripes, respectively. Furthermore, we use shadowed areas to show the waves of the main Covid 19 variants: *20E* (yellow), *Alpha* (rose), *Delta* (green) and *Omicron* (white zone after *Delta* variant). Before the vaccination campaign, the different waves are only affected by the containment policies with the circulation of predominant Wuhan variant in USA and Japan, while in UK the second and the third waves onsets correspond with the wave of the *20E* and *Alpha* variants, respectively. After the start of the vaccination campaign, at t_v_ = 350 days, despite periods with fairly high transmissibility rate (R_t_ ≥ 1), the spread of the pandemic has been effectively limited by the massive vaccination capable of slowing down the pandemic kinetics while maintaining T_d_ on high values, and avoiding the fall in the supercritical phase.

In UK this was accompanied by a strong suppression of the daily fatalities while Japan has been able to keep low the number of deaths with Df always < 1, thanks to the different “Covid zero” containment policy. However, in all three countries, the latest Omicron variant was found to be sufficiently dangerous to bring the spread of the pandemic back to the supercritical regime, thereby piercing the vaccine defense.

### Recurrent waves of Covid-19 in United States, United Kingdom and Japan

**Figure 3:**
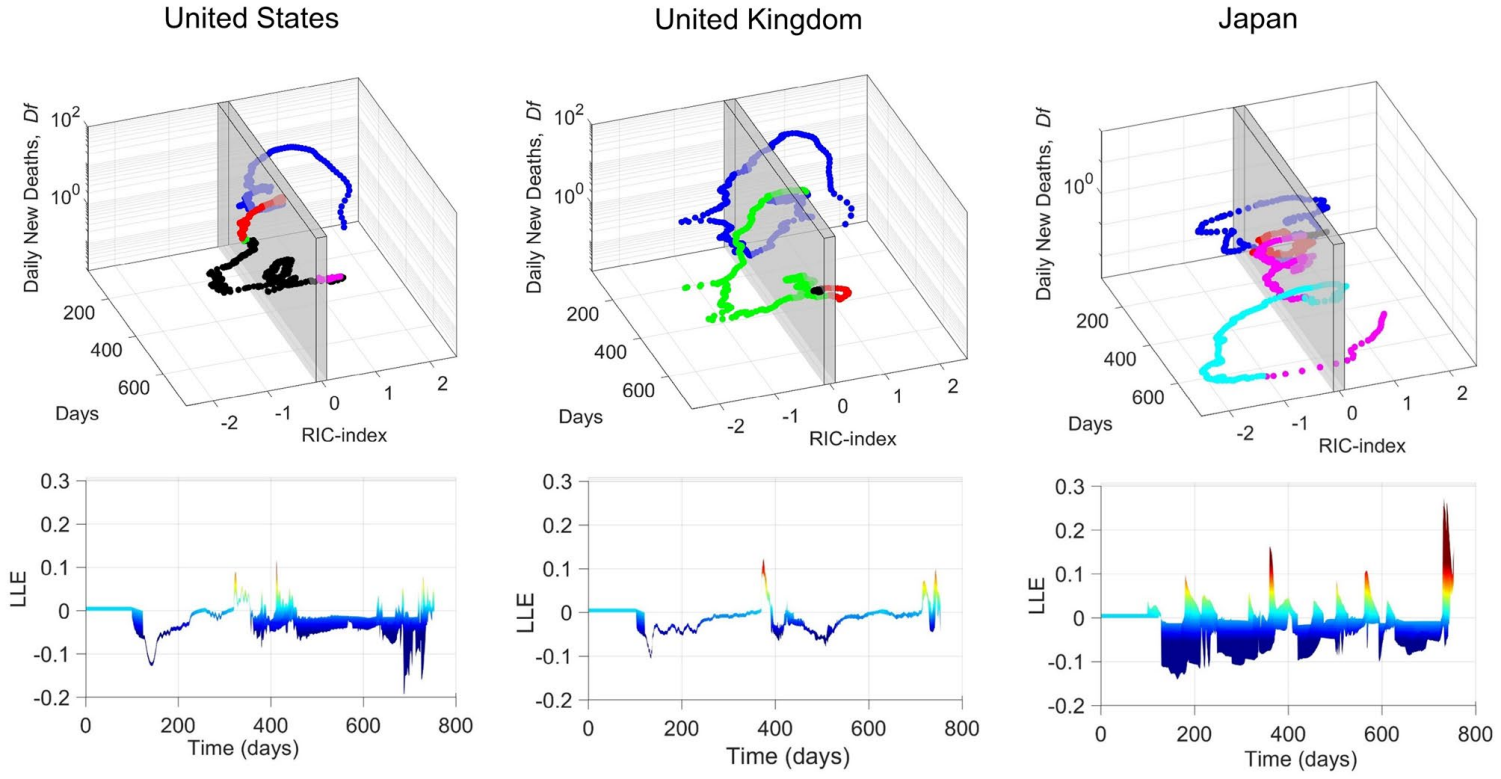
3D helicoid vortex and Lyapunov Maps of epidemic spreading from 15/03/2020 to 28/01/2022 in United States, United Kingdom and Japan. In the upper panels we show the 3D orbit of the Covid-19 vortex over two years where the daily new deaths per million population and the *RIC-index* are measured in days starting from the 75 day of the year 2020. The gray shaded slice represents the critical crossover at the zero *RIC* index, which separates the *supercritical* (*RIC* index > 0) and the *subcritical* regime (*RIC* index < 0). The lower panels represent the Lyapunov maps. The discontinuity points, where the LLE integrated along the different initial ranges change sign becoming positive, correspond to times in which the helicoid vortex change color. Non-positive LLE values imply convergent trajectories independent by the initial conditions of the phenomenon.

The pandemic waves in USA, UK and Japan have been visualized by helicoid vortices in the (*Df*, *RIC* index, time) phase space shown in Fig. 3, as it was shown in Fig. 1d for the entire world. In these vortices the different waves associated with the crossover from the convergent to the divergent character of the time evolving *Df*(*RIC*, time) orbits are determined not only by the vaccination campaign, as in the world case, but also by the appearing of Covid 19 variants with different lethality and transmissibility. This mechanism produces more crossovers in the Lyapunov maps, which identify convergent to divergent transitions, showed by different colored section of the trajectories on the helicoid vortices. In Japan, where the “*Covid zero*” policy was enforced, the largest number of waves driven by the dynamics of the infection spreading is observed.

**Figure 4:**
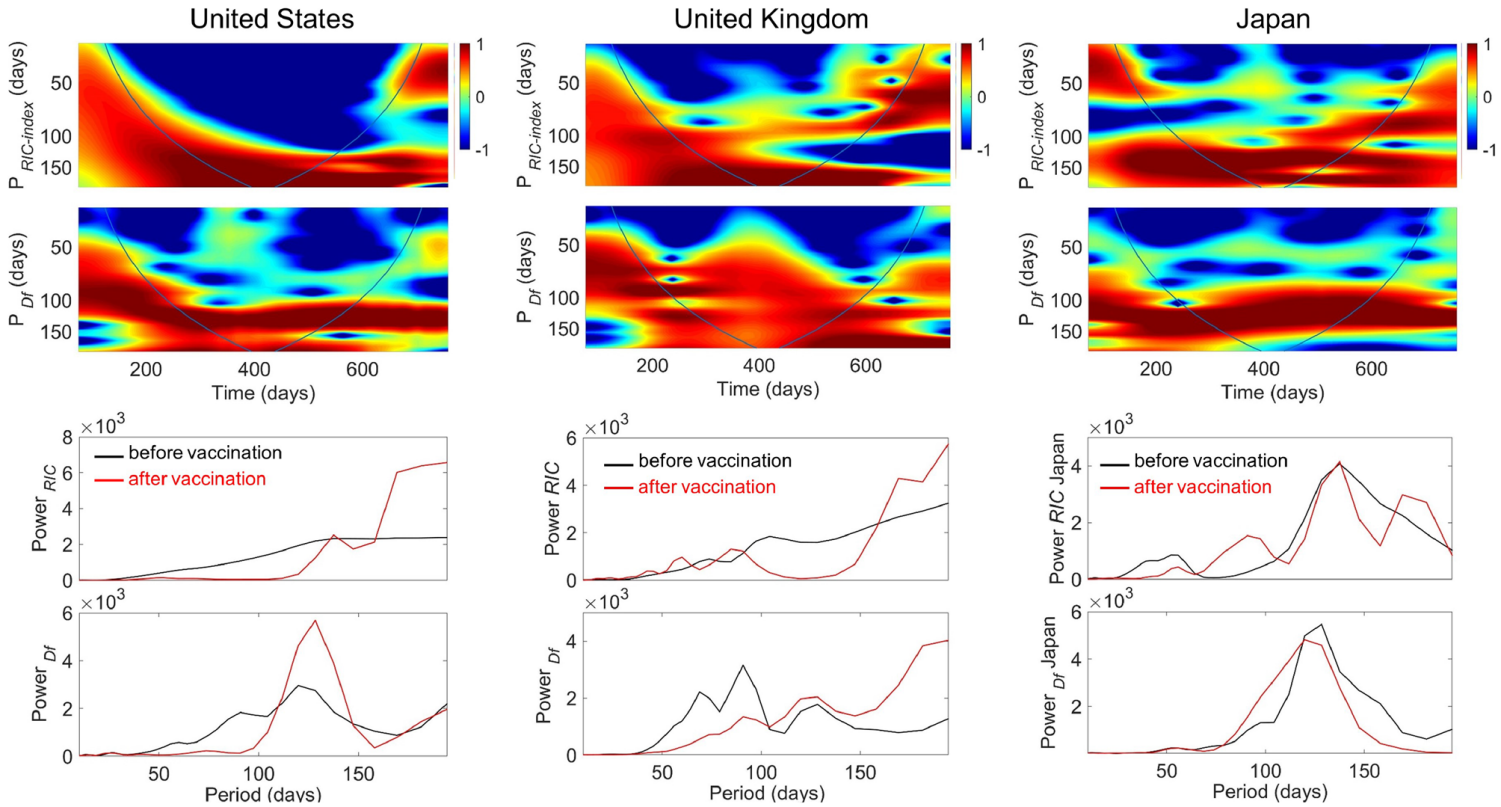
Wavelet and Fourier Transform of epidemic spreading in United States, United Kingdom and Japan from 15/03/2020 to 28/01/2022. The Covid-19 spreading is visualized vs. time by wavelet transform giving the Local Wavelet Power Spectrum (LWPS) of the *RIC* index and the daily deaths per million population *(Df)* time series in the upper panels. The wavelet analysis decomposes the spreading waves hidden in time series data at different scales. Power is color-coded from − 1 (blue) to 1 (red). In the lower panels we show the Global wavelet power spectrum (GWPS) of the period of epidemic periodic travelling waves for infections measured by the *RIC* index and the new deaths, before the vaccination campaign for *t* < *t*_*v*_ (black lines) and after the onset of the vaccination campaign for *t* > *t*_*v*_ (red lines).

The time–frequency decomposition obtained with the wavelet analysis^39,40^ is shown in Fig. 4 where are plotted the Local (LWPS) and Global (GWPS) Wavelet Power Spectra, of the selected countries. As in the world data, we observe an intrinsic different periods composition between infection and fatality waves. We still find in the fatality waves that the dominant period is ~ 130 days both before and after the vaccination campaign. However, this is accompanied by shorter periods in the fatality waves in UK and USA. Both short and long periods are lacking in the Japanese fatality waves, where the number of fatalities has been kept at low level with *Df* < 1, while we clearly distinguish two short periods of 68 and 90 days in the fatality waves for UK. These short periods disappear during the vaccination campaign in 2021 and a long period component (~ 180 days) become dominant. The spreading phenomenon in Japan points a key result of our analysis: the clear increase of the weight of the long (170 days) and the short (85 days) periods. The latter could be interpreted in the infection waves during the vaccination campaign as the second harmonic of the first.

## Discussion

We have monitored the evolution of the Covid-19 pandemic by analyzing its contagiousness and severity as a function of time from 15/03/2020 to 28/01/2022. The contagiousness is measured by the *RIC-index*, a combination of the effective reproduction number and the doubling time, while the hazardousness and severity are given by the daily deaths per million people. The pandemic waves have been visualized in a 3D space parameter where the fatalities number is correlated to the *RIC-index* and time. We find a helicoid vortex where the stability and unpredictability of trajectories have been studied through the *Lyapunov Map* built by the time dependent Largest Lyapunov Exponents of the reconstructed space made of the 3D orbits evolving day per day. The detection of any possible crossover from negative to positive LLE gives the instability zones corresponding to the onset of new pandemic waves. Identification of the periods of the pandemic waves has been obtained by wavelet transform of the *RIC* index and the daily deaths per million people time series. We used this approach to demonstrate the effect of the vaccination campaign on the pandemic periods, finding a common typical period of about 130 days, while during the vaccination campaign all periods are elongated. However, we observe an intrinsic difference between infection and fatality waves pointing to a non-trivial variation of the lethality due to different gene variants. Further work is in progress to monitor this difference after the vaccination, related with the appearing of the new variants and the duration of effectiveness of Covid.19 vaccine.

In summary, we have shown that the non-linear time evolution of the fatalities number and the *RIC-index* in the chaotic vortex describe the pandemic phenomenon and give the tools needed for a prompt response to the appearing of new waves triggered by new variants of the coronavirus. Its use to control the containment policies may provide early warning to critical situations and at the same time a quantitative measure of the slowing down of the rate of spreading of the virus and its variants while the vaccination campaign is running.

## Methods

### Calculations of T_d_, R_t_

All methods were performed in accordance with the relevant guidelines and regulations. To extract the time-dependent doubling time T_d_^21^ as described in^24^ and the time-dependent reproductive number R_t_ from^41^ all data on epidemic spreading and severity from 15/03/2020 to 28/01/2022 have been taken from the recognized public database *OurWorldInData*^42^ while the variants evolution data have been taken from the public database *CoVariants*^43^.

### Wavelet analysis

The wavelet transform is used to perform a time-scale decomposition of the data using functions (wavelets) that narrow when high-frequency features are present and widen on low-frequency structures. We used the Morse wavelet function^39,40^ decomposition, which provides the localization in both time and frequency. This function is well suited for investigating the temporal evolution of non-stationary and transient time series, as typically epidemics are. The outputs of the wavelet transform of the time series is the local wavelet power spectrum (LWPS), which allows for inspection of time–frequency distribution and detection of predominant signal components for particular time periods. Recently, this approach has been widely used in applied mathematics^44^. LWPS of the *RIC-index* and of the daily deaths per millions of populations (*Df*) time series are presented in Fig. 3. Global wavelet power spectrum (GWPS) of both *RIC-index* and daily deaths per millions of populations time series have been calculated by averaging the LWPS across time, giving standard Fourier transform, but in two different time windows: for t < t_v_ and for t > t_v_, i.e., before and after the beginning of the vaccination campaign. Traveling waves in the epidemic generates a peak in the power spectra. The wavelet transform has been performed, after smoothing accomplished using Gaussian windows on 14 days, for avoiding higher frequencies due to artefacts typically corresponding to data collection during public holidays and weekends.

### Lyapunov map

The Largest Lyapunov Exponents (LLE) of the *RIC* index and *Df* time series are calculated employing the algorithm by Rosenstein et al.^35^ after reconstructing the state space from experimental data record. The original time series data and its time-delayed copies determine the topological structure of the dynamical system^36–38^:

2 $$Y = \left[ {X\left( t \right), X\left( {t + T } \right), \ldots, X\left( {t + \left( {d - 1} \right)T} \right)} \right]$$

where *Y* is the reconstructed *d*-dimensional state vector, *X(t)* is the observed variable, *T* is a time lag, and *d* is the embedding dimension. A Largest Lyapunov exponent, in a Lyapunov map^35–38^ is calculated for a set of dynamic *Df* versus *RIC* index trajectories starting with different initial ranges and iteratively increased by one day up to a discontinuity point, where LLE goes from a negative to a positive value. These points, where a convergent trajectory becomes divergent, are called *discontinuity points*. As LLE become positive, we set these *discontinuity points* as initial instants of new series. The iteration of this procedure allows us to visualize and identify the *discontinuity points* that are independent from the different initial ranges in the calculations. The initial ranges have been chosen from a minimum of 28 days, that is a minimum value to reconstruct the state vector, up to the minimum main period found by the WT analysis (typically ~ 60 days). The quantitative determination of the discontinuity points on the helicoid 3D trajectory is made by finding the discontinuity points in the averaged profile of LLE along the used initial ranges in the map. As for the WT analysis, the Lyapunov Map is calculated on time series smoothed on Gaussian windows of 14 days, for avoiding higher frequencies due to the data collection artefacts mentioned above.

### Statement

The data reported in this paper do not report experiments involving live animals on live vertebrates and/or higher invertebrates or research that involves organs/tissues procured from prisoners.

## Data Availability

Relevant raw data, are freely available on the recognized public database https://ourworldindata.org/coronavirus-source-data and https://covariants.org/. All data generated or analysed during this study are included in this published article. Further information on the current study are available from the corresponding author on reasonable request.
